# Effects of Estrogen, an ERα Agonist and Raloxifene on Pressure Overload Induced Cardiac Hypertrophy

**DOI:** 10.1371/journal.pone.0050802

**Published:** 2012-12-05

**Authors:** Christina Westphal, Carola Schubert, Katja Prelle, Adam Penkalla, Daniela Fliegner, George Petrov, Vera Regitz-Zagrosek

**Affiliations:** 1 Charité – Universitätsmedizin Berlin, Institute of Gender in Medicine, Berlin, Germany; 2 Center for Cardiovascular Research, Berlin, Germany; 3 Bayer Pharma, Wuppertal, Germany; 4 German Heart Institute, Berlin, Germany; Max-Delbrück Center for Molecular Medicine (MDC), Germany

## Abstract

The aim of this study was to investigate the effects of 17β-estradiol (E2), the selective ERα agonist 16α-LE2, and the selective estrogen receptor modulator (SERM) raloxifene on remodeling processes during the development of myocardial hypertrophy (MH) in a mouse model of pressure overload. Myocardial hypertrophy in ovariectomized female C57Bl/6J mice was induced by transverse aortic constriction (TAC). Two weeks after TAC, placebo treated mice developed left ventricular hypertrophy and mild systolic dysfunction. Estrogen treatment, but not 16α-LE2 or raloxifene reduced TAC induced MH compared to placebo. E2, 16α-LE2 and raloxifene supported maintenance of cardiac function in comparison with placebo. Nine weeks after induction of pressure overload, MH was present in all TAC groups, most pronounced in the raloxifene treated group. Ejection fraction (EF) was decreased in all animals. However, 16α-LE2 treated animals showed a smaller reduction of EF than animals treated with placebo. E2 and 16α-LE2, but not raloxifene diminished the development of fibrosis and reduced the TGFβ and CTGF gene expression. Treatment with E2 or 16α-LE2 but not with raloxifene reduced survival rate after TAC significantly in comparison with placebo treatment. In conclusion, E2 and 16α-LE2 slowed down the progression of MH and reduced systolic dysfunction after nine weeks of pressure overload. Raloxifene did not reduce MH but improved cardiac function two weeks after TAC. However, raloxifene was not able to maintain EF in the long term period.

## Introduction

Heart failure (HF) is one of the most common causes of death worldwide. One important precursor for HF is myocardial hypertrophy (MH), which occurs in response to increased afterload, e.g. in pressure overload due to aortic valve stenosis [1]. After a pathologic stimulus like pressure overload, the heart responds first with compensatory MH with preserved systolic function. Thus, the early stage of MH is characterized by an increase in wall thickness without a loss of cardiac function. In later stages, chronic stress causes the dilation of the left ventricle (LV) and a loss of systolic or diastolic function, usually measured as a reduced ejection fraction (EF) or elevated end-diastolic pressure or an increase in ventricular stiffness [2], [3]. A further consequence of permanent pressure overload is the proliferation of fibroblasts, an increase in collagen synthesis, and remodeling of the extracellular matrix (ECM) [4].

Clinical manifestations of MH and HF differ between women and men. Survival in HF is better in women than in men [5]. Women with HF exhibit more often diastolic dysfunction with small ventricles and thickened walls [6], [7], whereas men suffer mainly from systolic dysfunction [8], [9]. In aortic stenosis induced pressure overload, in the presence of similar pressures and transvalvular gradients, women develop more concentric MH than men with better maintained myocardial function [2]. There is less profibrotic gene expression in women [10] and less induction of markers of pathological hypertrophy [11] In animal models of pressure overload and HF, sexual dimorphisms and a role for estrogen in the reduction of mortality in animal models with HF was described earlier [12], [13]. In a model with ovariectomized female mice, the treatment with E2 reduces left ventricular hypertrophy four and eight weeks after TAC [14].Treatment with 17β-estradiol (E2) attenuates myocyte hypertrophy as well as gene expression of the hypertrophic markers *atrial natriuretic peptide* (ANP) and *beta myosin heavy chain* (βMHC) after transverse aortic constriction (TAC) [15]. Treatment with E2 also leads to a reduction of fibrosis in animal models of MH and myocardial infarction (MI) [16], and influences the collagen [17] and *matrix metalloproteinase 2* (MMP2) expression [18], [19]. On the other hand, higher mortality rates due to E2 treatment are described in animal models both of MI and TAC [20]. This higher mortality may have extracardiac causes: different clinical trials like the Heart and Estrogen/Progestin Replacement Study (HERS) and the Women’s Health Initiative (WHI) show a higher incidence for thrombosis, breast cancer as well as cardiovascular diseases in the E2 treated patients [21], [22].

The effects of E2 are mainly mediated by the estrogen receptors (ER) alpha (ERα) and beta (ERβ). Both affect MH and fibrosis [23]. To dissect potential beneficial and harmful effects transmitted by E2 and ERα and ERβ in humans and animals in different tissues and organs, selective estrogen receptor modulators (SERMs) have been developed. The treatment with the selective ERα agonist 16α-LE2 led to a reduction of perivascular fibrosis and cardiac hypertrophy in aldosterone salt-treated rats that develop hypertension, MH, and HF [24]. SERMs like raloxifene act as ER agonists in the heart and bone tissue but as antagonists in the uterus and breast. Raloxifene shows a fourfold higher affinity to the ERα compared to ERβ. [25] In an animal model for transverse aortic constriction, raloxifene reduced cardiac dysfunction and the development of myocardial hypertrophy after a short time period, four weeks, after TAC [26].

The aim of our study was the evaluation of ERα-specific effects in the development of MH. In order to contribute to the understanding to the contradictory results in different studies, we compared the effects of E2, the ERα specific agonist 16α-LE2, and raloxifene on cardiac hypertrophy, systolic function, and fibrosis in a long term model of pressure overload-induced cardiac hypertrophy. We hypothesize, that the treatment with E2, 16α-LE2, and raloxifene prevent the development of myocardial hypertrophy and the loss of cardiac function to a different extent. To test this hypothesis, we analyzed ovariectomized female mice, which were treated with E2, 16α-LE2 or raloxifene.

## Results

### Treatment with E2 or 16α-LE2 Increased the Uterus Weight

As described before [14], [24], [27], treatment with E2 or 16α-LE2 but not placebo or raloxifene led to an increase of uterus weight (E2: 120.9±13.97 mg; 16α-LE2: 137.9±8.60 mg; placebo: 14.54±2.23 mg; raloxifene: 15.77±1.30 mg). E2 or 16α-LE2 treated animals, which did not show a significant increase in uterus weight, were excluded from the study.

### Treatment with E2 and 16α-LE2 Attenuated the Progression of Hypertrophy and the Loss of Systolic Function

**Figure 1:**
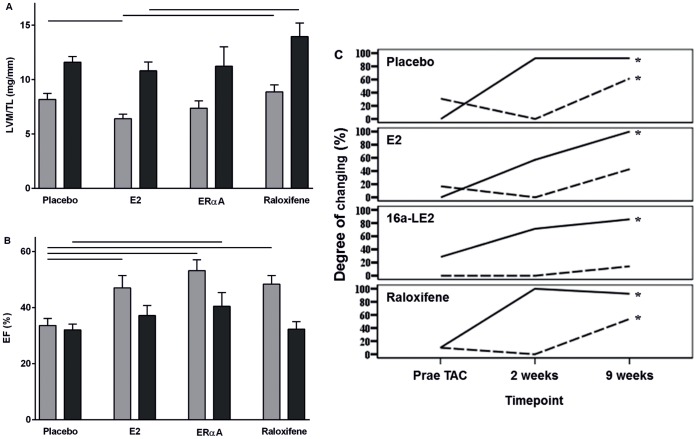
Development of cardiac hypertrophy and LV function two weeks (grey bars) and nine weeks (black bars) after TAC. (A) Left ventricular mass to tibia length ratio (LVM/TL) was significantly reduced by E2 treatment compared to placebo or raloxifene treated animals two weeks after TAC. After nine weeks, raloxifene treated animals showed a significant higher LVM/TL than E2 treated mice. ANOVA post-hoc Scheffé; p-value p<0.05. (B) Ejection fraction (EF) showed a significant reduction of left ventricular function two weeks after TAC in placebo treated mice compared to all treated groups. Nine weeks after TAC surgery exclusively mice with ERα agonist treatment showed a significant higher EF. ANOVA post-hoc Scheffé; p-value p<0.05. (C) Factor analysis underlined the positive influence E2 and 16α-LE2 treatment on the progression of MH and loss of LV function. Broken line: factor *LV function*; solid line: *LV morphology*; Friedman test; p-value p<0.05.

TAC led to a significant increase in LV hypertrophy expressed as left ventricular mass/tibia length (LVM/TL) in placebo treated mice after two and nine weeks (Table 1). Two weeks after TAC, E2 reduced the hypertrophic response (Figure 1A). At this time point, 16α-LE2 treatment led to a similar reduction of LVM/TL compared to placebo that did however not reach statistical significance. Raloxifene treatment did not affect MH. After nine weeks, neither E2 nor 16α-LE2 nor raloxifene had an effect on LVM/TL.

**Table 1:** LV hypertrophy and function 2 and 9 weeks after sham or TAC surgery.

	LVM/TL		EF	
	2 weeks	9 weeks	2 weeks	9 weeks
**ShamOVX+ShamTAC**	5.92±0.15	6.33±0.27	42.11±3.04	41.17±3.26
**ShamOVX+TAC**	7.75±0.37#	10.72±0.54#	38.09±2.07	32.96±3.06#
**OVX+ShamTAC**	5.99±0.22	5.96±0.23	44.72±2.25	44.35±3.03
**OVX+TAC**	8.17±0.55*	11.59±0.52*	33.57±2.55*	32.02±2.08*

T-test;

# p<0.05 vs. ShamOVX+ShamTAC.

* p<0.05 vs. OVX+ShamTAC.

Chronic pressure overload and progression of LV hypertrophy are related to left ventricular dysfunction. Induction of pressure overload by TAC led to a decrease of EF after two and nine weeks in placebo treated animals (Table 1). E2, 16α-LE2, and raloxifene prevented the decrease in EF in the early stage after two weeks (Figure 1B). After nine weeks, only 16α-LE2 and E2 treatment protected against the loss of systolic function, whereas raloxifene could not prevent the decrease of EF.

In order to analyze the effects of E2, 16α-LE2 and raloxifene on the progression of hypertrophy and systolic dysfunction simultaneously, we used *factor analysis* including all relevant echocardiographic parameters followed by Friedman test for time-dependent changes during the experiment (Figure 1C). [28] The *factor analysis* is a statistical model, which allows a simplification of complex relations by correlation of all variables. High correlation of two or more variables, led to the generation of one new variable, which was called *factor*. The factor analysis identified two new factors. Factor 1 was associated with LV dilatation quantified by an increase in left ventricular internal diameter (LVID; factor loading 0.974) and a worsening of contractile performance quantified by decrease in EF (factor loading −0.911) and fractional shortening (FS; factor loading −0.905). Since factor 1 was strongly associated with these two major determinants of LV function, it was used as marker for time-dependent changes in *LV function* (broken line, Figure 1C). Factor 2 was aggregated from the wall thicknesses (IVS; factor loading 0.917) and end-diastolic left ventricular posterior wall thickness (PWd; factor loading 0.891), and LVM/TL (factor loading 0.851) and was used as an indicator of time-dependent changes in *LV morphology* (solid line, Figure 1C). *LV morphology* was altered under conditions of pressure overload. As described by factor 2 there was strong evidence for hypertrophic remodeling (wall thickening and increase in LV mass) in all animals over the whole experimental period. However the time course of hypertrophy development varied between E2, 16α-LE2 and raloxifene treated animals compared to the placebo. Regarding LV morphology raloxifene treated animals showed a very similar hypertrophic remodeling to placebo. Hypertrophy development in the first two weeks after TAC was maintained mainly by wall thickening (Table 2). After that there was no noteworthy change in LV morphology, but a strong decrease in LV function. In contrast, E2 and 16α-LE2 treatment led to a slighter slope in the LV morphology curve two weeks after TAC and thus slowed the development of hypertrophy. Simultaneously E2 and 16α-LE2 treatment prevented adverse changes of *LV function* nine weeks after TAC in comparison to the raloxifene treated group.

**Table 2:** Echocardiographic parameter for all measured time points.

		placebo	E2	16α-LE2	raloxifene
**IVSd (mm)**	**before TAC**	0,69±0,02	0,61±0,01	0,74±0,03	0,69±0,04
**LVIDd (mm)**		3,93±0,05	3,88±0,12	3,98±0,14	4,15±0,06
**PWd (mm)**		0,60±0,02	0,58±0,03	0,63±0,03	0,65±0,02
**LVIDs (mm)**		3,36±0,06	3,02±0,18	2,96±0,18	3,26±0,12
**IVSd (mm)**	**two weeks after TAC**	0,98±0,03	0,84±0,03*	0,90±0,07	1,10±0,06† ‡
**LVIDd (mm)**		3,76±0,05	3,73±0,08	3,69±0,07+	3,82±0,09
**PWd (mm)**		1,03±0,05	0,82±0,05*	0,88±0,07/	0,94±0,04
**LVIDs (mm)**		3,06±0,06	2,86±0,12	2,69±0,10*	2,91±0,12
**IVSd (mm)**	**nine weeks after TAC**	1,06±0,03	1,02±0,06	1,11±0,10	1,21±0,06*
**LVIDd (mm)**		4,38±0,11	4,17±0,13	4,04±0,09	4,67±0,14† ‡
**PWd (mm)**		1,06±0,04	0,96±0,05	1,02±0,13	1,02±0,05
**LVIDs (mm)**		3,73±0,13	3,44±0,17	3,25±0,17*	3,97±0,17‡

* p<0,05 vs. Placebo;

† p<0,05 vs. E2;

‡ p<0,05 vs. ERα agonist

**Figure 2:**
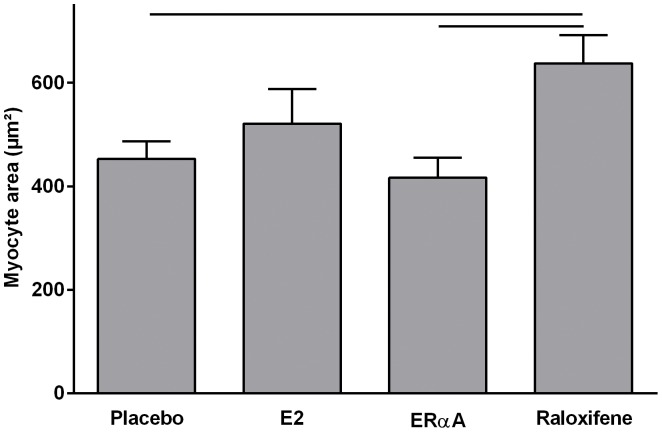
Myocyte hypertrophy after nine weeks of chronic pressure overload. According to LVM/TL raloxifene treated animals developed a significant larger myocyte area compared to placebo and 16α-LE2 treated animals. ANOVA post-hoc Scheffé; p-value p<0.05.

To investigate the mechanisms of hypertrophy we analyzed myocyte area immunohistologically by wheat germ agglutinin (WGA) staining after nine weeks. E2 or 16α-LE2 treatment did not reduce cellular hypertrophy (Figure 2). The treatment with raloxifene even enhanced the myocyte area compared to placebo treated animals.

### Reduction of Molecular Markers of Hypertrophy and Fibrosis by E2 and 16α-LE2

E2 and 16α-LE2, but not raloxifene reduced the expression of the hypertrophic marker brain natriuretic peptide (BNP) in TAC animals. The hypertrophic markers ANP and βMHC (Table 3) showed an expression pattern similar to BNP. The expression of ANP, BNP as well as βMHC strongly correlated with LVM/TL (ANP: r = 0.738; BNP: r = 0.459, βMHC: r = 0.785) and EF (ANP: r = 0.561, BNP: r = 0.610, βMHC: r = 0.584).

**Table 3:** Gene expression of hypertrophic markers and matrix genes.

	ANP/HPRT	BNP/HPRT	βMHC/HPRT	TGFβ1/HPRT	TGFβ2/HPRT
**placebo**	2.50±0.54	2.72±0.28	2.85±0.43	1.14±0.22	1.05±0.24
**E2**	1.11±0.25*	1.65±0.38*	1.09±0.27*	0.71±0.14	0.52±0.13
**16α-LE2**	1.38±0.25	1.00±0.16*	1.18±0.47*	0.68±0.20	0.73±0.24*
**raloxifene**	2.72±0.54†	2.06±0.31‡	2.87±0.62‡	0.44±0.08*	0.77±0.24

* p<0,05 vs. Placebo;

† p<0,05 vs. E2;

‡ p<0,05 vs. ERα agonist.

**Figure 3:**
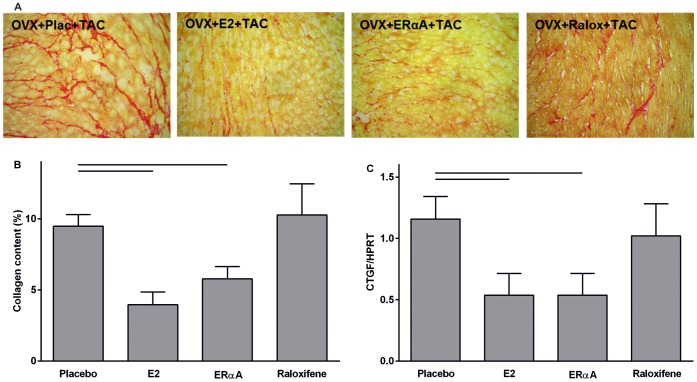
Remodeling of the extracellular matrix after nine weeks of pressure overload. (A) Representative pictures of collagen staining (Sirius Red) suggest a higher overall collagen content in placebo and raloxifene treated mice compared to E2 and 16α-LE2 treated animals. (B) Quantification of fibrosis score showed a significantly lower level of collagen in mice with E2 and ERα agonist; ANOVA post-hoc Scheffé; p-value p<0.05 (C) Comparable with the collagen content *connective tissue growth factor* (CTGF) gene expression was significantly lower in mice with E2 or 16α-LE2; ANOVA post-hoc Scheffé; p-value p<0.05.

E2 or 16α-LE2 treatment led to a significant reduction of the overall fibrosis score (reflecting collagen content calculated by Sirius Red staining) in LV, but raloxifene did not Figure 3A and 3B). Further, E2 and 16α-LE2 treatment decreased relative mRNA expression of *connective tissue growth factor* (CTGF), whereas raloxifene had no effect (Figure 3D). Parallel changes were observed for *transforming growth factor beta* (TGFβ) 1 and 2, but did not achieve statistical significance (Table 3).

### Treatment with E2 and 16α-LE2 Decreased Survival Rate after TAC

**Figure 4:**
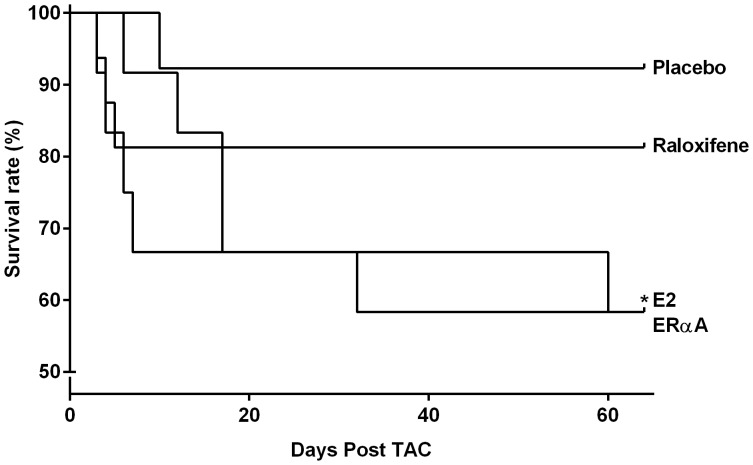
Chronic pressure overload induced mortality. Highest survival rate was observed in placebo treated animals after TAC (92.3%; 1 out of 13). Treatment with E2 or 16α-LE2 led to a significant lower survival rate compared to placebo groups (both groups: 58.3%; 5 out of 13). Log rank Test; p<0.05.

Animals with pressure overload had a mortality rate below 10% during the experimental period if treated with placebo. The treatment with E2 or 16α-LE2 increased the mortality significantly to approximately 40% during nine weeks and occurred mainly during the first two weeks (Figure 4).

## Discussion

We compared the effects of different strategies of hormone therapy in an early and late stage of myocardial hypertrophy (two and nine weeks after TAC). The treatment with E2 and the ERα agonist 16α-LE2 decelerated the development of cardiac hypertrophy, diminished left ventricular dysfunction, and reduced collagen accumulation. However, we also observed a significant lower survival rate in these groups. The treatment with raloxifene slowed the loss of systolic function in the early stage after two weeks of pressure overload, but not after nine weeks.

MH occurs as an early consequence of pressure overload. After induction of pressure overload, MH first develops as compensated MH, as myocyte hypertrophy without induction of fibrosis. Later, it turns fast into pathological MH with fibrosis which is poorly reversible and is accompanied by a decrease in cardiac function. There is an ongoing discussion whether moderate or so called adaptive or compensated MH is needed after induction of pressure overload to maintain wall tension in a near normal range and to prevent further deterioration, or whether MH is always an ominous prognostic sign [26], [29], [30].

In our study, LVM/TL increased in ovariectomized female mice two weeks after TAC by 30%. At this time point the decrease in systolic function already reached 90% of its decrease after nine weeks, suggesting that MH was no more compensated. An earlier study in a comparable model showed no decrease in systolic function at this time point, but these animals were not ovariectomized [31]. Thus, ovariectomy may have speeded up the functional decrease. In agreement with this hypothesis, E2 treatment inhibited the early increase in MH two weeks after TAC in the current study and diminished the decrease in cardiac function during the whole experiment. This is well in agreement with other reports that show suppression of pathological MH by E2 [14], [15]. 16α-LE2 and raloxifene did not affect MH but reduced the decrease in ejection fraction after two weeks. After nine weeks, a better systolic function in comparison with placebo was only maintained with 16α-LE2 treatment. In accordance with our findings the protective effects of 16α-LE2 to maintain cardiac function also have been shown in a model of SHR. [32] Based on our findings, there is the evidence that the hypertrophic phenotype was not affected by any compound until the end of the experiment, but treatment with E2 or the ERα agonist 16α-LE2 decelerated the development of MH in the early stage and limited functional decline during the whole experiment. The SERM raloxifene was described to mediate cardioprotective effects in a two weeks TAC model [26]. Accordingly, in the raloxifene treated group we observed a preserved EF two weeks, but not nine weeks after TAC. Furthermore, the four years clinical *Raloxifene Use for the Heart (Ruth)* trial Ruth [29] showed also no significant effects of raloxifene on the risk for cardiovascular events after 5.6 years of treatment.

Fibrosis, the accumulation of collagens, usually accompanies the development of pathological MH and loss of function. It contributes to a decrease of contractility. The removal of endogenous E2 in the ageing heart enhances the collagen accumulation [33]. A number of studies have generated evidence that E2 regulates collagen synthesis in a sex-specific manner and particularly decreases collagen synthesis in female hearts [10]. Our present study showed similar results. The E2 treatment decreased the total collagen content nine weeks after TAC. In line with this, Pedram et al. [34] observed a significant reduction of fibrosis after E2 treatment in ovariectomized wild type mice treated with angiotensin II. But, the authors hypothesized that ERα is not involved in the development of fibrosis. In contrast, in our experiments the ERα agonist 16α-LE2 reduced the development of fibrosis in a model of chronic pressure overload. Accordingly, it was shown, that the treatment with 16α-LE2 attenuates perivascular fibrosis in aldosterone salt-treated rats [24]. In addition, our own group showed that E2 inhibits MMP-2 expression via ERα in cardiac fibroblasts and lead to a more beneficial cardiac remodeling under stress conditions [19].

Supporting the effects on fibrosis and collagen synthesis, E2 and 16α-LE2 treatment reduced the CTGF gene expression significantly (Figure 3D). This growth factor is induced by TGFβ and contributes to the development of fibrosis [35], which expression was slightly diminished in all treated animals compared to placebo. Missing significant effects in TGFβ mRNA expression are probably due to the fact, that TGFβ is an early responsive gene [36] and may not be present at all desirable time points. It seems that the treatment with E2 or the ERα agonist inhibits CTGF and thereby profibrotic gene expression after chronic pressure overload. The underlying mechanisms need further investigation.

Different animal studies showed a reduction of the survival rate after treatment with E2 in MI as well as TAC models [14], [20]. In agreement with these, we observed a significant reduction of the survival rate in E2 and 16α-LE2 treated animals. We did not observe markers for HF in the dead animals (data not shown). However, high doses of estrogen are known to cause thrombotic events and this may have contributed to the high mortality with E2 treatment in our study. Further, uteri weights in the E2 and 16α-LE2 treated groups were excessively increased, well above physiological weights.

In conclusion, our data suggest a protective influence of E2 and 16α-LE2 on the heart under conditions of pressure overload. Both substances E2 and the specific ERα agonist 16α-LE2 decelerated the hypertrophic response of the left ventricle and prevented the loss of cardiac function until the end of the experiment. The protective effects of E2 and 16α-LE2 are also reflected by reduced fibrosis formation. Thus, pathways downstream of ERα may have the potential to be exploited for cardioprotection. In contrast, raloxifene had only positive effects after two weeks and was not able to prevent the dramatic loss of ejection fraction after nine weeks of pressure overload.

### Limitations of the Study

Serum levels of E2, 16α-LE2, and raloxifene have not been obtained. Uterus weight was chosen as marker of atrophy due to ovariectomy and an adequate response to E2 and 16α-LE2 treatment [37].

We did not measure fibrosis and profibrotic gene expression after two weeks because the study was designed as an end point study at nine weeks, when we expected a decompensated stage of MH.

## Methods

### Animals

Female C57BL/6J mice (Harlan Winkelmann, Germany) were housed under standard conditions on a12 h/12 h light/dark cycle in temperature controlled rooms. Animals were kept on a soy-free diet from the age of 3 weeks (Ssniff, Soest, Germany) and water ad libitum. Mice were anesthetized with Ketamine Hydrochloride (80 mg/ml)/Xylazine Hydrochloride (12 mg/ml) solution administered by intraperitoneal injection at a dose of 1 mg/kg for ovariectomy, pellet placement, and transverse aortic constriction (TAC). All animal procedures were performed in accordance with the guidelines of the Charité - Universitätsmedizin Berlin, and were approved by the Landesamt für Gesundheit und Soziales (LaGeSo, Berlin, Germany) for the use of laboratory animals (G0149/06) and followed the “Principles of Laboratory Animal Care” (NIH publication No. 86-23, revised 1985), as well as the current version of German Law on the Protection of Animals.

### Ovariectomy

At the age of seven weeks ovariectomy (ovx) was performed by a standard bilateral abdominal procedure.

### Estrogen Replacement

One week after ovx 90-day-release pellets containing 390 µg E2, 70 µg 16α-LE2, 22.5 mg raloxifene or placebo were implanted into the upper neck subcutaneously. All pellets were purchased from Innovative Research of America. Substances were provided by Bayer Schering AG, Berlin, Germany. At the end of the experiment, the uteri have been removed to check the responsiveness to hormone replacement therapy.

### Transverse Aortic Constriction

Animals underwent sham surgery (sham) or transverse aortic constriction (TAC) at the age of nine weeks by a recently described method [31]. After nine weeks, animals (n = 9–13 per group) were sacrificed and organs were harvested. Left ventricles were immediately frozen in liquid nitrogen and stored at –80°C until use.

### Echocardiographic Measurements

Echocardiographic measurements were done according to the standards established in human echocardiography and previously described. Measurements were performed the day before as well as two and nine weeks after TAC respectively sham surgery and left ventricular mass (LVM) and ejection fraction (EF) were calculated.

### Histology

Frozen cardiac tissues (n = 5 per group) were cut into 5 µm sections and stained with Sirius Red for evaluation of overall collagen content and calculation of the fibrosis score.

Myocyte size was measured with wheat germ agglutinin (WGA) staining (Fluorescein wheat germ agglutinin antibody, Vector Laboratories, Inc., Burlingame, CA, USA).

### Real Time Polymerase Chain Reaction (RT-PCR)

Gene expression was evaluated by Taqman RT-PCR using the Power SYBR®Green PCR Master Mix (ABI Applied Bioscience, Carlsbad, USA). The primer sequences used for real time PCR are: **ANP** (*atrial natriuretic peptide*) forward: 5′-CCT GTG TAC AGT GCG GTG TC, reverse: 5′-CCT CAT CTT CTA CCG GCA TC, **BNP** (*b-type natriuretic peptide*) forward: 5′-GCA CAA GAT AGA CCG GAT CG, reverse 5′-CAG GCA GAG TCA GAA ACT GGA; **βMHC** (*beta myosin heavy chain*) forward: 5′-CAA AGG CAA GGC AAA GAA AG, reverse: 5′-TCA CCC CTG GAG ACT TTG TC, **CTGF** (*connective tissue growth factor*) forward:. 5′-TTC CCG AGA AGG GTC AAG CT, reverse: 5′-TTG GGT CTG GGC CAA ATG T, **TGFβ1** (*transforming growth factor β 1*) forward: 5′-CCA AGG AGA CGG AAT ACA GG, reverse: 5′-GTT CAT GTC ATG GAT GGT GC; **TGFβ2 (** *transforming growth factor β 2*) forward: 5′-TAC TGC AGG AGA AGG CAA GC; reverse: 5′-TAG AAA GTG GGC GGG ATG **HPRT** (*hypoxanthine phosphoribosyltransferase 1*) forward: 5′-GCT TTC CCT GGT TAA GCA GTA CA, reverse: 5′-ACA CTT CGA GAG GTC CTT TTC AC.

The amount of target cDNA sample was calculated in relation to HPRT.

### Statistics

All data are demonstrated as means ± SEM and were assessed for normal distribution. Survival data were tested by Log-Rank test. All other data were tested by two-way ANOVA and post-hoc Scheffé using SPSS 17.0. To analyze changes in *LV morphology* and *LV function* during progression of hypertrophy the *factor analysis* was used. The *factor analysis* is a statistical model for dimension reduction of complex datasets. In this study it integrates per design time-dependent relations between multiple echocardiographic variables into less information rich factors, which we semantically interpreted as LV morphology and LV function. High correlation of two or more variables, led to the generation of one new variable, which was called *factor*. Friedman test [38] was used to reveal statistically significant changes within the factors over the whole experimental time course.

For all statistical analysis p values ≤0.05 were defined as statistically significant.
